# Emerging Genotype (GGIIb) of Norovirus in Drinking Water, Sweden

**DOI:** 10.3201/eid0912.030112

**Published:** 2003-12

**Authors:** Karin Nygård, Maria Torvén, Camilla Ancker, Siv Britt Knauth, Kjell-Olof Hedlund, Johan Giesecke, Yvonne Andersson, Lennart Svensson

**Affiliations:** * Swedish Institute for Infectious Disease Control, Solna, Sweden; † Karolinska Hospital, Stockholm, Sweden; ‡ University of Linköping, Linköping, Sweden

**Keywords:** Norwalk-like viruses, gastroenteritis, waterborne outbreak, norovirus

## Abstract

From May through June 2001, an outbreak of acute gastroenteritis that affected at least 200 persons occurred in a combined activity camp and conference center in Stockholm County. The source of illness was contaminated drinking water obtained from private wells. The outbreak appears to have started with sewage pipeline problems near the kitchen, which caused overflow of the sewage system and contaminated the environment. While no pathogenic bacteria were found in water or stools specimens, norovirus was detected in 8 of 11 stool specimens and 2 of 3 water samples by polymerase chain reaction. Nucleotide sequencing of amplicons from two patients and two water samples identified an emerging genotype designated GGIIb, which was circulating throughout several European countries during 2000 and 2001. This investigation documents the first waterborne outbreak of viral gastroenteritis in Sweden, where nucleotide sequencing showed a direct link between contaminated water and illness.

Viruses have emerged as important causes of foodborne and waterborne diseases in recent years, with numerous outbreaks associated with Norwalk viruses. This virus is the prototype in the genus *Norovirus,* family *Caliciviridae*, which includes a large number of genetically related strains associated with acute gastroenteritis. Longitudinal surveys have shown that caliciviruses and especially noroviruses are common causes of nosocomial and community-associated outbreaks of acute gastroenteritis worldwide (*1*–*5*). Norovirus-associated gastroenteritis is transmitted by the fecal-oral route. It occurs both as sporadic community cases and as large outbreaks in, for example, nursing homes, hospitals, schools, and ships. The outbreaks often are associated with ingestion of food or contaminated water. Norovirus-associated waterborne outbreaks (*6*) have been associated with contamination of septic tanks, industrial water system (*7*–*9*), and swimming water (*10*–*12*) as well as drinking contaminated drinking water (*13*–*18*).

We describe a waterborne outbreak caused by contaminated drinking water. While no pathogenic bacteria were found in collected samples, identical noroviruses belonging to genogroup II (GGIIb) were identified in both stool and water samples.

## Methods

### Outbreak Description

An outbreak of acute gastroenteritis occurred in a combined activity camp and conference center in Stockholm County from May to the end of June 2001. During the summer, the center caters to both overnight guests and daytime visiting groups. A separate cafe for outside visitors to the nearby beach is also on the premises. Environmental and microbiologic investigations were conducted to determine the source of the outbreak and implement control measures to stop the outbreak and prevent similar situations in the future.

### Environmental Investigation

The municipal environmental health unit was first contacted on June 12. The facilities were inspected, and water and food samples were collected. On June 15, the Stockholm County Council Department of Communicable Disease Control and Prevention was contacted, and the premises were reinspected on June 25 and July 3. Additional water samples were taken on several occasions during June and July.

### Microbiologic Investigation

#### Bacteriologic Investigation

A total of 11 stool specimens were collected (2 from staff and 9 from visiting guests) and cultured for bacterial enteropathogens, including *Salmonella, Shigella,* *Campylobacter,* and *Yersinia*. Ten water samples were examined for fecal coliforms, total coliforms, fecal streptococci, and sulphite-reducing clostridia. Seven food products were examined for aerobe microorganisms, enterobacteriaceae, enterococci, fecal coliforms, *Salmonella,* *Bacillus cereus*, C*lostridium perfringens,* coagulase-positive staphylococci, yeast, and mold. Approved standard laboratory methods were used for all bacteriologic investigations.

### Virologic Investigation

Stool samples were examined for norovirus by electron microscopy and reverse transcription–polymerase chain reaction (RT-PCR), as previously described (*4*,*19*,*20*). Briefly, viral RNA was extracted from 100 μL of a 10% stool suspension with the guanidine thiocyanate–silica extraction method (*21*) followed by RT-PCR with primer pair JV12/JV13, which yields a 326-bp product, located in the gene for RNA-dependent RNA polymerase.

Three water samples collected from the kitchen, the water works, and the public beach were tested for norovirus. These water samples were concentrated by a method slightly modified from Gilgen et al. (*22*). Briefly, 0.5 L of water was filtered through a positively charged 0.45-μm membrane (Zetapor, Millipore Corp., Bedford, MA) followed by virus elution from the membrane with 50 mM glycine–NaOH, pH 9.5, containing 1% beef extract as described (*16*). A Centricon-100 microconcentrator (Amicon, Millipore) was used for further concentration to 100 μL.

For the water samples, a nested PCR was used. RNA-extraction and first-round PCR were performed as described in this section. For the nested PCR, new inner primers were designed from alignment of sequences circulating in Sweden and sequences from the GenBank database. The inner primers were designated n12 (5′-TGG GAY TCM ACD CA-3′) and n13 (5′-CTT CAG ANA GNG CAC ANA GAG T-3′). These primers yield a 234-bp product.

### Nucleotide Sequencing

The PCR products from two human and two water samples were sequenced. The samples were sequenced from both directions by using primer pair n12/n13 (water samples) and primer pair JV12/JV13 (patient samples) by ABI Prism BigDye Terminator Cycle Sequencing Ready Reaction kit (Applied Biosystems, Foster City, CA) on an ABI 310 automated sequencer. Sequences from prototype strains of caliciviruses from the GenBank database were aligned with the sequences from patient and water samples. Programs from the PHYLIP program package (National Institutes of Health, Besthesda, MD) were used to construct the phylogenetic trees. SEQBOOT (NIH) was used for bootstrap resampling to produce 100 different datasets from the aligned sequences. From these datasets, phylogenies were estimated by DNAMLK (NIH). CONSENCE (NIH) was used to construct a consensus tree from the obtained data and to obtain bootstrap values. The tree was drawn with Treeview (Page RD. TREEVIEW, University of Glasgow, Glasgow, Scotland). The nucleotide sequence accession number assigned by GenBank is AY240939.

## Results

### Environmental Investigation

The activity camp, conference center, and nearby cafe were supplied with ground water from their own private wells, located at the premises. Six months before the outbreak, they had started to use water from two newly drilled wells located within 20 m of each other. Only chemical parameters had been analyzed before the new wells were put in use. The water from both wells was held in a common reservoir and was not disinfected before distribution. According to personnel at the camp, the wells were approximately 80 m deep, and the soil layer was 18 m at the location of the wells. A third well was drilled at the same time and located close to the other two but was not put in use. Previously, water had been obtained from an old well located further away from the facilities. Since this old well had limited capacity, and sometimes its water was not potable, new wells with enough capacity to fulfill increased demands had been drilled. For practical and economical reasons, the new wells had been placed closer to the center facilities.

Sewage from the camp was connected to the community system and was transported to the nearest sewage treatment facility. The sewage pipes were old, and personnel reported that on several occasions problems with the capacity of the system had occurred. In April 2001, a blockage of the overflow in the low-pressure-system well, located near the kitchen facilities, occurred, and sewage had spilled out on the ground. On this site, located approximately 100 m from the ground water wells, the rock was covered by only 1–2 m of soil. Sewage had also overflowed on the ground near the kitchen in the autumn 2000 because of a stoppage in the sewage pipeline connection to the community system.

### Epidemiologic Investigation

Approximately 200 people contracted gastroenteritis after consuming tap water. They had clinical symptoms of vomiting, diarrhea, abdominal pain, and fever (mostly a combination of these symptoms). Duration of symptoms varied from several hours to 2 to 3 days. The first known cases of illness occurred in a group of adults participating in a 1-day conference on May 31. Of 16 persons (all adults), 8 became ill (attack rate 50%) with gastrointestinal symptoms. Nearly 2 weeks later (June 9–10), a school class with 28 pupils (8–13 years of age) arrived for an overnight stay; approximately half became ill (attack rate 50%) with similar symptoms. The following day (June 10), the first participants of a sport-training camp arrived. The camp lasted for 10 days, during which a total of 150 children (9–12 years of age) and 20 adults stayed at the facilities in three overlapping periods. The first cases of illness in this group occurred the day after arrival; approximately 100 persons became ill (attack rate 58%). During the next 2 weeks, several more guests and visiting groups reported illness after visiting the center; some of these persons had not eaten but had just drunk the center’s tap water. Two of these groups were children (8–13 years of age); the attack rate in both groups was 40%. The outbreak was not controlled until the facilities closed for >1 week in the end of June. Some of the personnel working at the center also reported gastrointestinal symptoms, including one of the kitchen personnel, who became ill on June 13 and was taken off duty.

### Control Measures

On the first visit, general recommendations regarding kitchen hygiene and cleaning of the environment were given. When the results of the first water samples were ready, additional recommendations on boiling all water used for drinking and food preparation were given. At the same time, the environment was thoroughy sanitized. In spite of these measures, new cases continued to occur, so the facilities were closed for >l week at the end of June to interrupt possible continuous transmission among guests. After this measure, no new cases occurred. Different alternatives to prevent similar situations in the future were discussed, and the decision was made to close the wells and connect to the municipal water supply.

### Microbiologic Investigation

None of the stool samples collected from the two staff or nine visitors were positive for *Salmonella, Shigella, Campylobacter,* or *Yersinia,* nor were any viruses other than calicivirus found by electron microscopy. Of the 11 samples examined by norovirus-specific PCR, 8 had an amplified PCR product of the expected size. No foodborne pathogens were found in any of the food items investigated. The first samples were collected from tap water in the kitchen on June 12 and water collected from the water works on June 18 showed strong indication of fecal contamination (Table). Samples collected from the wells 1 and 3 on June 20 and 27 showed evidence of fecal contamination, as did sampling of well 2 in July (Table). Water samples from the tap in the kitchen and the water works, collected on June 18, were positive for norovirus with a nested PCR and showed evidence of fecal contamination (Table). The water samples collected from the beach were negative for norovirus. PCR amplicons from two visitors (samples collected at different time points) and the two positive water samples were sequenced and compared. The strains were identical to each other and identical to strain “Gothenburg” (Figure) and had 97%-98% nucleotide identity to Spanish GGIIb strains (AJ487474,AJ487794, AJ487795, AJ487789, AJ487794) (*23*).

**Table:** Results from bacteriologic analysis of water samples, Sweden, 2001

Place	Date (2001)	Heterotrophs/ mL (2d)	Coliforms/ 100 mL	*E. coli^a^/* 100 mL	Sulphite-reducing clostridia/100 mL	Fecal streptococci /100 mL
Tap water, kitchen	06/12	80	140	47	-	-
Water works	06/18	690	100	32	<1	2
Tap water, kitchen	06/18	530	130	40	<1	1
Well 1	06/20	>300	430	>100	-	-
Well 2	06/20	>300	1	<1	-	<1
Well 3	06/27	2,100	19	1	-	-
Old well	06/27	1,100	630	22	-	-
Storm water	06/27	2,000	190	3	-	-
Well 2	07/03	1,300	160	6	-	-
Beach	07/17	16,000	1	-	-	-

^a^ *Escherichia coli.*

**Figure:**
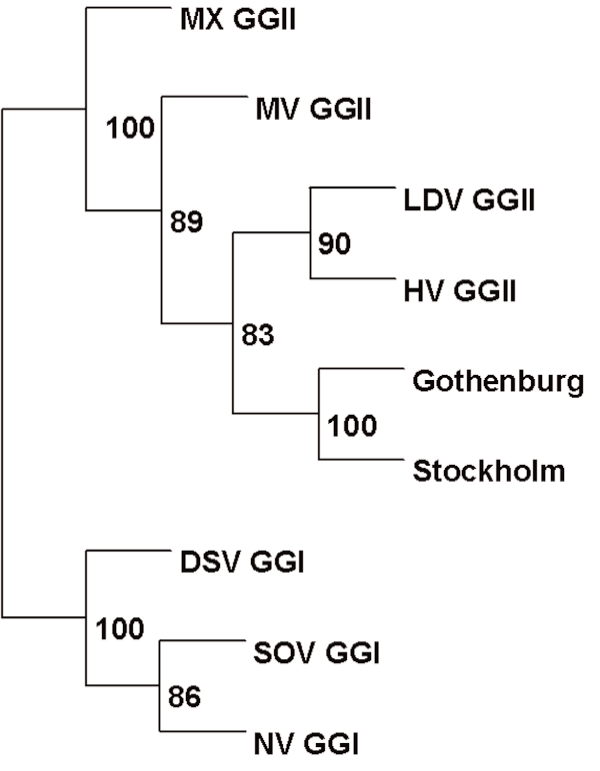
Phylogenetic tree based on a 198-nt region of the gene coding for RNA-dependent RNA-polymerase (located in ORF1), showing patient and water samples and some prototype strains of calicivirus from the GenBank database (accession no.: MX, Mexico U22498; MV, Melksham X81879; HV, Hawaii U07611; LDV, Lordsdale X86557; DSV, Desert Shield U04538; SOV, Southampton L07418; NV, Norwalk M87661; Gothenburg AF365989). Bootstrap values are given in percentage at the nodes.

## Discussion

We describe an epidemiologic and microbiologic investigation of a waterborne outbreak in which at least 200 persons became ill after staying at a combined activity camp and conference center in the Stockholm area. A large number of daytime visitors to the beach and nearby cafe may also have become ill, so the actual number of cases has likely been underestimated. The visitors in different groups did not eat the same food items, and some visitors did not eat any food. Several of the short-stay visitors consumed only camp tap water, which was fecally contaminated. The source of illness was drinking water obtained from ground water wells that had been contaminated by sewage. Person-to-person transmission and transmission through contaminated surfaces probably contributed to the rapid spread among the overnight visitors. While no pathogenic bacteria were found in water or stool samples, norovirus belonging to genogroup II with identical nucleotide sequence in the polymerase region was obtained from both stool and water samples. The strain was identical to strain Gothenburg, previously identified in Sweden and belonging to the emerging genotype cluster GGIIb. These strains have circulated in several European countries during 2000 and 2001 (*23*). While the GGIIb outbreak in this study was associated with contaminated water, previously reported GGIIb strains have been associated with school, nursing home, and rural village outbreaks (*23*). That all were identified during 2000 and 2001 further supports the hypothesis of an emerging strain or cluster of strains.

The drinking water was obtained from deep ground wells close to the cafe. Before the outbreak, this cafe had had problems with low pressure in its well, which caused blockage of the sewage system. As a consequence sewage spilled out and lead to contamination of the environment. At the contamination site, the soil was only 1–2 m deep, and cracks in the rock may have facilitated migration of microorganisms from the sewage to the ground water. Norovirus can migrate through soil and contaminate well water and cause gastroenteritis outbreaks (*7*,*24*).

One possible explanation for the protracted duration of the outbreak could be a continuous leak from the sewage system, which would have caused persistent contamination of the environment. The ill persons staying at the facilities might have contributed to increased viral load in the sewage, and problems with the sewage collection system would then have further aggravated contamination of the water supply. Another possibility was that the water initially caused the outbreak, but person-to-person spread contributed to the continuous transmission.

The low infectious dose of norovirus readily allows transmission through environmental contamination and aerosols. Boiling the water used for drinking and food preparation was recommended. Since the risk for transmission through aerosols generated when showering with possibly contaminated water is not well established, no recommendations were made in this regard. Another problem was how to decontaminate bed linen and other fabrics. Washing at high temperatures is the recommended procedure to eliminate viral contamination. However, if the water used for washing is contaminated, the rinsing process may lead to recontamination of the fabrics. We recommended boiling or heating water for washing to >90°C in the presence of detergents.

This outbreak illustrates some problems related to private water supply. In Sweden, approximately 15% of the population has a private water supply, and the extent of gastrointestinal illness related to water is not clearly identified. Problems with person-to-person transmission of noroviruses are well known; however, risks related to exposure through contact with contaminated water and environment through vomit and aerosols are not well established.

In summary, detecting identical virus in both drinking water and stool specimens from ill persons strongly indicated that norovirus was the principal pathogen of this outbreak. Nucleotide sequence analysis identified a norovirus designated GGIIb (*23*).
